# Potential Alteration of Statin-Related Pharmacological Features in Diabetes Mellitus

**DOI:** 10.1155/2021/6698743

**Published:** 2021-03-26

**Authors:** Habibeh Mashayekhi-Sardoo, Stephen L. Atkin, Fabrizio Montecucco, Amirhossein Sahebkar

**Affiliations:** ^1^Department of Pharmacodynamics and Toxicology, School of Pharmacy, Mashhad University of Medical Sciences, Mashhad, Iran; ^2^Weill Cornell Medicine Qatar, Doha, Qatar; ^3^IRCCS Ospedale Policlinico San Martino Genoa-Italian Cardiovascular Network, 10 Largo Benzi, 16132 Genoa, Italy; ^4^First Clinic of Internal Medicine, Department of Internal Medicine, University of Genoa, 9 Viale Benedetto XV, 16132 Genoa, Italy; ^5^Biotechnology Research Center, Pharmaceutical Technology Institute, Mashhad University of Medical Sciences, Mashhad, Iran; ^6^Applied Biomedical Research Center, Mashhad University of Medical Sciences, Mashhad, Iran; ^7^Halal Research Center of IRI, FDA, Tehran, Iran; ^8^Polish Mother's Memorial Hospital Research Institute (PMMHRI), Lodz, Poland

## Abstract

**Objective:**

Type 2 diabetes mellitus is a chronic metabolic disease caused by insulin resistance or insulin deficiency resulting in elevated blood glucose levels. Poorly controlled diabetes is associated with the development of cardiovascular disease and dyslipidemia. 3-Hydroxy-3-methylglutaryl coenzyme A (HMG-CoA) reductase inhibitors (statin) are an important class of therapeutic agents used to control hyperlipidemia and prevent cardiovascular disease in diabetic and nondiabetic patients. Since the effect of diabetes on the pharmacokinetics and pharmacodynamics of drugs and toxins has been shown, the aim was to review previous studies on the efficacy of statins such as atorvastatin, simvastatin, pravastatin, pitavastatin, fluvastatin, and rosuvastatin in clinical and preclinical studies in both diabetic and nondiabetic groups.

**Method:**

For this purpose, Web of Science, PubMed, Scopus, and Google Scholar databases were reviewed, and related English articles published until October 2020 were included in this review article.

**Results:**

The findings revealed that diabetes affected statin effectiveness through changes in pharmacokinetic parameters such as clearance and biotransformation biomarkers at mRNA and protein levels. Plasma and serum concentrations of statins were accompanied by alteration in cellular activities including oxidative stress, Akt inhibition, and endothelial nitric oxide synthase (eNOS) and phosphorylation that were reflected in changes in the adverse drug reaction profile of the differing statins.

**Conclusion:**

Given that dyslipidemia frequently accompanies diabetes and statin therapy is common, more clinical studies are needed regarding the effects of diabetes on the effectiveness of these drugs.

## 1. Introduction

Type 2 diabetes mellitus (DM) is a chronic metabolic disease caused by insulin resistance or insulin deficiency resulting in elevated blood glucose levels. The prevalence of DM is reaching epidemic proportions with major patient and health economic consequences. Poorly controlled diabetes is associated with increased morbidity and mortality with atherosclerotic cardiovascular disease being the major cause of death [1]. Elevated glycated hemoglobin, hypertension, dyslipidemia, low physical activity, and smoking are related independent risk factors associated with atherosclerotic cardiovascular disease incidence [2].

Dyslipidemia is characterized by increased serum triglyceride (TG) levels and diminished high-density lipoprotein (HDL) levels, along with increased small dense LDL [3]. Although the exact mechanism of diabetic dyslipidemia pathogenesis is not clear, insulin resistance is a suggested mechanism in this progress. This leads to raised free fatty acid (FFA) release from insulin-resistant fat cells with an elevated flux of FFA to the liver and along with adequate glycogen stores in this tissue; this induces the production of TG, thereby promoting the Apolipoprotein B (ApoB) and VLDL cholesterol secretion. Correspondingly, the impaired insulin activity for the inhibition of FFA release from insulin-resistant fat cells increases the production of hepatic VLDL cholesterol associated with the degree of hepatic fat accumulation [4–7].

The 3-hydroxy-3-methylglutaryl coenzyme A (HMG-CoA) reductase inhibitors (also known as statins) are drugs that are effective treatment for lowering the low-density lipoprotein- (LDL-) cholesterol levels in patients with dyslipidemia [8]. In recent years, there has been an increasing literature on the antioxidant and cardioprotective effects of statins through lowering LDL-cholesterol levels in diabetic and/or nondiabetic patients [9, 10]. However, statin use and an increased risk of diabetes onset are a concern though impact of statins on insulin levels and insulin resistance are unclear [11]. The major available statins such as atorvastatin, simvastatin, pravastatin, pitavastatin, fluvastatin, and rosuvastatin show differing efficacies and drug interactions.

A considerable literature has been published on the effects of DM on the pharmacokinetics and pharmacodynamics of drugs, natural agents, and toxins in both clinical and animal models. These studies revealed that DM may affect statin absorption followed by changes in the blood flow in muscle and subcutaneous adipose tissue, micro- and macrovasculature, and gastric emptying, in the distribution by glycosylation or movement of plasma proteins. This leads to alterations in the plasma protein binding of statins through alteration of the enzymes/transporters related to drug biotransformation such as cytochrome P450 (CYP) enzymes and the glutathione-S-transferase (GST) superfamily. In addition, statin excretion is affected because of nephropathy followed by alterations in the angiotensin-converting enzyme and micro- and macrovascular structure of the kidney [12–14]. The published data regarding the effect of DM on the pharmacodynamics of drugs or toxins are rare and are limited to immunosuppressive and cardiovascular drugs [15].

Since DM leads to cardiovascular disease with associated dyslipidemia, statin use is common and widespread, but the effect of DM on statin therapy pharmacokinetics and pharmacodynamics is poorly documented; therefore, this narrative review was undertaken on the effect of DM on differing statins and their side effects in both clinical and preclinical studies.

## 2. Search Strategy

In this study, relevant keywords including “diabetes”, “diabetes mellitus”, “adverse drug reaction”, “toxicity”, “pharmacokinetics”, “toxicokinetic”, “pharmacodynamic”, “mechanism”, “statin”, “HMG-CoA reductase inhibitor”, “dyslipidemia”, and “hyperlipidemia” were searched in the Web of Science, MEDLINE (PubMed), Scopus, and Google Scholar to retrieve articles that examined the effects of diabetes on the pharmacokinetics, pharmacodynamics, and side effects of different types of statins. Besides, the bibliography of each article was reviewed to identify suitable/pertinent studies in order to include all relevant reports published until October 2020. All included studies were in English. The review studies were excluded from assessing.

## 3. Result and Discussion

### 3.1. Atorvastatin

Diabetic patients who take HMG-CoA reductase inhibitors due to decreased clearance of statin lactone are at the risk of myotoxicity development. Generally, atorvastatin prescribed as atorvastatin acid is biotransformed by cytochrome P450 3A4 (CYP3A4) in the liver and gastrointestinal system that creates active para-hydroxy (p-OH) and ortho-hydroxy (o-OH) metabolites along with inactive lactone metabolites. Atorvastatin lactone is very lipophilic and possesses a higher 83-fold affinity to CYP3A4 compared to atorvastatin acid. Consequently, atorvastatin lactone that is an intermediate metabolite contributes to atorvastatin acid clearance [12, 15]. Dostalek et al. [12, 15] determined the effect of DM on the pharmacokinetics of atorvastatin acid and its chief metabolite in diabetic and nondiabetic patient renal transplant recipients where they determined the biotransformation of atorvastatin acid and atorvastatin lactone in human liver microsomal fractions of diabetic and nondiabetic donors. They showed that the plasma concentration of atorvastatin lactone was higher than that of atorvastatin acid; however, its clearance was notably lower than that of atorvastatin acid over a 24 hr period. The population pharmacokinetic model for atorvastatin lactone was a one-compartment model as well as for atorvastatin. The *in vitro* findings revealed that (1) CYP3A4 metabolizes atorvastatin lactone and (2) atorvastatin acid concentration did not differ, but atorvastatin lactone concentration was higher than that in hepatic microsomal fractions of diabetic donors. This showed that DM leads to a decrease in clearance and an increase in plasma concentration of atorvastatin lactone as an atorvastatin acid metabolite. This finding is important to guide adjusting the atorvastatin dose in diabetic patients with other comorbidities.

Many studies have shown a reduction in coronary atheroma plaque development through statin therapy, but this is less in DM patients with acute coronary syndrome (ACS). In a prospective study, atorvastatin given to diabetic or nondiabetic patients with ACS revealed the same changes in LDL-C (ΔLDL) levels in both groups. In diabetes patients, a significant correlation existed between ΔLDL and percent atheroma volume change (ΔPAV), but there was no relationship between ΔPAV and ΔLDL in nondiabetic patients. Collectively, ACS patients with diabetes in comparison to nondiabetes patients showed weaker coronary plaque regression. Further intensive lipid-lowering treatment is necessary for the DM patients to address the relationship between ΔLDL and ΔPAV [16].

Using diabetic rats induced by streptozotocin (STZ), the decrease in serum atorvastatin concentrations through an enhancement in biotransformation enzymes in the liver and elevation of atorvastatin clearance was investigated. Their findings suggested that dosage adjustment is very important in diabetic patients who are hyperlipidemic and these patients may require higher dosages of atorvastatin compared to hyperlipidemic patients without diabetes (H. [17]).

Diabetes affects harmful advanced glycation end products (AGEs) that alter protein activities leading to histopathological damage in kidneys such as glomerular basement membrane (GBM) thickening and elevated fractional mesangial volume, along with podocyte abnormalities, resulting in diabetic nephropathy (DN) and cardiovascular disease. Investigation of the effect of atorvastatin and diabetes on the CYP3A2 mRNA expression and enzymatic function in the kidney of male Wistar diabetic rats (50 mg/kg STZ, b.w., i.p.) and their testosterone metabolism showed significant upregulation of CYP 3A2 mRNA in the rat kidney tissue and increased 6b-hydroxytestosterone metabolite production. Atorvastatin by decreasing the Blood Urea Nitrogen (BUN) and creatinine (Cr) contributes to the improvement of nephropathy, as well as atorvastatin by the reduction in oxidative stress. The effect of atorvastatin and diabetes on the CYP3A2 renal metabolism indicated that the dose adjustment for CYP3A2 substrates is necessary [18].

Atorvastatin is metabolized with CYP3A4/Cyp3a in human/rat, and the possible effects of diabetes on CYP enzymes were evaluated using atorvastatin (oral (10 mg/kg) and intravenous (2 mg/kg)) in diabetic rats. The findings reported a considerable enhancement in atorvastatin clearance following both oral and intravenous treatments resulting in a significant reduction of area under the plasma concentration time curve (AUC) in diabetic rats. There was an increase in metabolism and uptake of atorvastatin in accord with the upregulation of hepatic protein Cyp3a and Oatp2 expression and their increased activities. Thus, this study suggested that upregulation of hepatic Cyp3a and Oatp2 proteins in diabetic rats results in a reduction in the plasma concentration of atorvastatin [19, 20].

Fan et al. [21] investigated whether or not acute or chronic treatment with atorvastatin plus ischemic postconditioning (IPost) would afford protection in nondiabetic and diabetic rats. The animals in the acute study received 50 *μ*mol/L atorvastatin in the time of reperfusion accompanied by IPost, and in the chronic study, the animals were treated with 10 mg/kg atorvastatin daily for 2 weeks plus IPost. In both time-dependent studies, the animals underwent 30 min ischemia followed by 120 min reperfusion. The findings provided evidence that IPost did not reduce the size of infarct and/or meliorate contractile dysfunction of diabetic rat hearts. In the acute atorvastatin study plus IPost, the treatment limited infarct size and protected contractile dysfunction in two nondiabetic and diabetic groups, and activation of Akt and endothelial nitric oxide synthase (eNOS) expression increased the protective benefits of atorvastatin; however, in the chronic statin study plus IPost, the infarct size and myocardial dysfunction did not change. Together, these findings indicated that acute atorvastatin administration plus IPost had increased heart protection whilst chronic statin administration plus IPost did not.

Acute kidney injury (AKI) due to the production of reactive oxygen species (ROS), renal vasoconstriction, hypoperfusion, hypoxia, and hypotension results in renal ischemic-reperfusion injury (IRI). Atorvastatin is considered an antioxidant and anti-inflammatory medication though the impact on renal ischemic-reperfusion (I/R) healing in diabetic or nondiabetic rat models required clarification. In an experiment, the animals of each (diabetic or nondiabetic rat) group received 10 mg/kg of atorvastatin and then underwent bilateral renal ischemia for 45 min followed by 24 hr of reperfusion. Nondiabetic rats improved and healed the glomerulus and proximal and distal tubules of the kidney, whilst in the diabetic rats, mild improvement of renal cortices, dilation of tubules, lining cell vacuolation, and hemorrhagic exudate in interstitial tissue were seen. The CD44 and caspase-3 expression, oxidative stress, and kidney function biomarkers were improved in both groups. The study concluded that atorvastatin may have beneficial and protective effects on renal I/R injury in rats with or without diabetes [22].

As noted above, atorvastatin is the substrate of CYP3a and OATP transporters; Wang and colleagues [23] designed a semiphysiologically based pharmacokinetic (semi-PBPK) model to predict the association between function and CYP3a expression alteration and atorvastatin transporters in rats with induced diabetes. The findings demonstrated that diabetes resulted in an elevation in hepatic CYP3a and OATP1b2 expression and a reduction in CYP3a and OATP1a5 expression in the intestine. They suggested that reduced activity and expression of CYP3a in the intestinal border of diabetic rats were attenuated through enhancement of function and expression of CYP3a and OATP1b2 in the liver.

Hepatotoxicity is one of the common side effects of atorvastatin: 40 mg/kg of atorvastatin was the leading cause of mortality in diabetic rats induced by STZ and a high-fat diet, whilst 10 mg/kg and 20 mg/kg of atorvastatin caused severe hepatotoxicity. *In vitro* findings showed that atorvastatin treatment led to severe cytotoxicity in diabetic rat hepatocytes. There was a significant positive correlation between hepatic Cyp3a and solute carrier organic anion transporter family member 1B1 (SLCO1B1) expression and action in diabetic rats and hepatotoxicity. Upregulations of SLCO1B1 and CYP3A4 in liver hepatocellular carcinoma (HepG2) cells triggered ROS production and then hepatotoxicity. These findings suggested that in general, the hepatic Cyp3a and SLCO1B1 upregulation in diabetic rats induced atorvastatin hepatotoxicity due to ROS generation [19, 20].

### 3.2. Simvastatin

Statins possess pleiotropic activities including cardiovascular benefits and anti-inflammatory effects. DM is an inflammatory disease along with increased oxidative stress and a risk factor for cardiovascular disease (CVD); therefore, a study was carried out to compare the potency of the anti-inflammatory effects of simvastatin (a single 10 mg/day for six months) in diabetic and nondiabetic patients with end-stage renal disease (ESRD) on chronic hemodialysis (HD). The findings showed that simvastatin lowered the total lipid profile in both groups, but the decrement was greater in diabetic patients in comparison to nondiabetic patients. Nevertheless, the reduction of inflammation and endothelial dysfunction markers including interleukin-6 (IL-6), soluble intercellular adhesion molecule-1 (sICAM-1), and soluble vascular cell adhesion molecule-1 (sVCAM-1) was greater in nondiabetic compared to diabetic patients, indicating the anti-inflammatory time properties of simvastatin. These findings recommended the prescription of the higher doses of simvastatin to get the parallel outcomes in the different groups [24].

In a study assessing adverse cardiovascular events after ACS in diabetic patients with low-density lipoprotein cholesterol 50 to 125 mg/dL, with the administration of 40 mg placebo versus simvastatin, it was shown that diabetic patients who had a former myocardial infarction (MI) and revascularization experienced more non-ST segment elevation acute coronary syndromes (NSTE ACS) in comparison to nondiabetic patients. In addition, the median of low-density lipoprotein cholesterol in diabetic patients was lower, and diabetic patients showed a decrease in myocardial infarction and ischemic stroke [25].

A retrospective propensity score-matched cohort study looked at the efficacy of 20 mg/kg simvastatin in patients with or without diabetes and assessed the major adverse cardiovascular events (MACE). This study showed an increased incidence of mortality in diabetic compared with nondiabetic patients; however, this difference was not significant. The main leading causes of death were as follows: multiple organ failure (MOF) following pneumonia or septic shock, unchecked bleeding, and a range of cancers. It is notable that the risk of MACE incidence in patients with diabetes was more than that in patients without diabetes [26].

Evaluation of simvastatin (20 mg/kg, PO, or 2 mg/kg, i.v.) and its hydrolysate simvastatin acid pharmacokinetics in the STZ model of diabetic rats showed that in diabetes both types of administration resulted in a significant reduction in simvastatin and simvastatin acid AUC. Furthermore, the examination of Cyp3a activity and simvastatin metabolism accompanied by Cyp3a1 and Oatp2 expression and activity in hepatic microsomes of diabetic rats demonstrated significant elevation of Cyp3a function and simvastatin metabolism along with enhancement of Cyp3a1 and Oatp2 mRNA expression. These results indicated that diabetes through the upregulation of hepatic Cyp3a activity and Oatp2 expression increased the simvastatin and simvastatin acid metabolism [27].

### 3.3. Fluvastatin

Diabetic patients undergoing percutaneous coronary intervention (PCI) and coronary artery bypass grafting (CABG) have generally poorer outcomes. Statin use may improve the coagulation system and endothelial dysfunctions in patients with diabetes. A study on the effect of fluvastatin on the relative survival rate of MACE (reintervention procedure (CABG and repeat PCI), nonfatal myocardial infarction, and cardiac death) over 3- to 4-year duration showed that in diabetic patients on fluvastatin, there was not any association between the presence of diabetes and elevated risk of MACE. Fluvastatin decreased the MACE risk by 51% and the incidence of atherosclerotic vascular events [28].

Rocha et al. [29] developed stereoselective pharmacokinetics of oral fluvastatin (5 mg/kg) as a racemic mixture of (–)-(3S,5R)- and (+)-(3R,5S)-enantiomers in diabetic and nondiabetic rats and showed the alteration of pharmacokinetics of fluvastatin because of diabetes in a stereoselective manner.

### 3.4. Pravastatin

Further experimental evidence on atherosclerosis development through uncontrolled diabetes and hyperlipidemia was investigated using pravastatin. Pravastatin effects on hepatic transporter mRNA expression in diabetes were investigated; 5 mg/kg pravastatin was administered to diabetic rats and then the pharmacokinetics of pravastatin was evaluated. The results showed that the expression of oatp2 was increased, promoting pravastatin transportation into hepatocytes leading to lower plasma concentrations of pravastatin. Correspondingly, because of decreased multidrug resistance-associated protein 2 (MRP2) expression, the transportation of pravastatin to bile was low causing decreased biliary excretion [30].

In animal studies, treatment of diabetes is performed by inducing rats with 10 mg/day pravastatin for 28 days, whilst pravastatin concentrations were increased in rat serum; it was reported that pravastatin may prevent the onset of diabetes, but it did not have any effect on blood glucose [31].

### 3.5. Pitavastatin

Pitavastatin is a lipophilic statin with pleiotropic activities and with a LDL-cholesterol-lowering effect. To investigate its effects in acute myocardial infarction (AMI) and diabetes, 2 mg/day of pitavastatin was prescribed to consecutive AMI patients (diabetic and nondiabetic) who underwent PCI after showing the AMI symptom. Angiographic findings were determined at 6 months and the clinical outcomes at 12-month duration. Diabetes patients were older, and they suffered from hypertension, left main lesion, and multivessel coronary artery disease (CAD), previous PCI, and lower LV ejection fraction compared to nondiabetic patients. After 12 months of follow-up, total death, TVR-MACE, total MACE, and repeat PCI were seen more commonly in diabetic patients after univariate analysis. In the multivariate analysis after baseline adjustment, diabetes was recognized as an independent predictor of TVR-MACE [32].

### 3.6. Comparative Studies of Statins

A study reported that pitavastatin (1-2 mg/d) and simvastatin (10-20 mg/d) administration to elderly patients with or without type 2 diabetes and hypertension showed similar decrease in lipids and liver dysfunction in both groups. Furthermore, they stated that pitavastatin was effective and safe with few mild adverse reactions in elderly patients with or without diabetes for CAD treatment [33].

A recent prospective study was aimed at comparing the relationship between inpatient statin (any type or dosage) therapy and stroke recurrence in Chinese diabetic and nondiabetic patients with an acute ischemic stroke history. The patients had no history of statin use. No relationship between statin therapy and stroke recurrence was seen between three-month and one-year follow-up duration in patients with diabetes, whereas statin therapy in patients without diabetes showed a remarkable reduction in stroke recurrence between three-month and one-year follow-up. It may be of benefit to have additional intensive therapy plus statin use in diabetic patients with acute ischemic stroke [34].

The beneficial effects on plaque stabilization in diabetes patients in comparison to nondiabetes were evaluated by researchers who looked at a lipid index and minimum fibrous cap thickness (FCT) over 1 year in diabetic and nondiabetic patients with CAD who were taking statins and assessed by serial optical coherence tomography imaging. The results suggested that statin therapy considerably increased the minimum FCT but significantly decreased the lipid index in two groups with no differences between them. Moreover, the same vascular response to treatment with a statin was seen in patients with or without diabetes [35].

A retrospective cohort study in Spain, 2006-15, was aimed at evaluating the association between statin use and reduction in atherosclerotic CVD and mortality in patients with and without diabetes aged 75 years or more who were statin-naïve or had just started statin therapy. They reported that in nondiabetic patients, statin therapy was associated with a paradoxical increase in the atherosclerotic CVD incidence, whilst the converse was seen in diabetes with a decrease in atherosclerotic CVD incidence and mortality [36].

## 4. Summary and Conclusion

The current review gives an overview and critique of the effect of DM on the pharmacokinetics, pharmacodynamics, and adverse drug reaction of different types of statins in both clinical and preclinical models. The findings revealed that diabetes affected statin effectiveness through changes in pharmacokinetic parameters such as clearance and biotransformation biomarkers at mRNA and protein levels (Table 1). Plasma and serum concentrations of statins were accompanied by alteration in cellular activities including oxidative stress, Akt inhibition, and endothelial nitric oxide synthase (eNOS) and phosphorylation that were reflected in changes in the adverse drug reaction profile of the differing statins. Given that dyslipidemia frequently accompanies diabetes and statin therapy is common, more clinical studies are needed regarding the effects of diabetes on the effectiveness of these drugs.

## Figures and Tables

**Table 1:** Evidence from clinical studies and animal models of statin pharmacological findings in diabetes mellitus.

Study design	Study sample	Samples with DM no./total no. (%)	Statin	Dose and duration	Recruitment time	Assessment of clinical response	References
*In vivo*	Nondiabetic and diabetic patients	20 nondiabetic and 32 diabetic renal transplant recipients	Atorvastatin acid	—	—	↓ clearance and ↑ plasma concentration of atorvastatin lactone as atorvastatin acid metabolite	(M. Dostalek et al., 2012)
*In vitro*		Human liver microsomal fractions obtained from 12 nondiabetic and 12 diabetic donors					
Prospective study	ACS patients with diabetes	126 ACS patients with DM (*n*: 13) or without DM	Atorvastatin	—	9–12 months	A considerable correlation between ΔLDL and ΔPAV in diabetes patients No relationship between ΔPAV and ΔLDL in nondiabetic patients	[16]
Animal study	Male Wistar diabetic rats (50 mg/kg STZ, i.p.)	Atorvastatin-treated diabetic (*n*: 6)	Atorvastatin	10 mg/kg, orally	Days 29-35 after diabetes induction	↓ serum atorvastatin concentration ↑ biotransformation enzymes ↑ atorvastatin clearance	(H. [17])
		Atorvastatin-treated nondiabetic (*n*: 6)					
Animal study	Male Wistar diabetic rats (50 mg/kg STZ, i.p.)	Nontreated diabetic (*n*: 8), atorvastatin-treated diabetic (*n*: 8)	Atorvastatin	10 mg/kg, orally	Two weeks	↓ CYP3A2 mRNA expression	(Hassan [18])
		Atorvastatin-treated nondiabetic (*n*: 8), control (*n*: 8)					
Animal study	Male Wistar diabetic rats (35 mg/kg STZ, i.p.)	*N*: 5	Atorvastatin	10 mg/kg: oral 2 mg/kg i.v.	Single dose	↑ atorvastatin clearances after both oral and intravenous treatments ↓ AUC in diabetic rats	[19, 20]
Cellular study	Primary hepatocytes of diabetic rats			0.1, 0.5, 2, 10, 40, and 100 *μ*M			
Animal study	Male Wistar diabetic rats (40 mg/kg STZ, i.p.), nondiabetic rats	Acute atorvastatin study plus IPost	Atorvastatin	50 *μ*mol/L	During reperfusion (120 min)	Limitation of infarct size and retrieval of contractile dysfunction in two nondiabetic and diabetic groups Activation of the Akt and eNOS expression ↑ protective benefits of atorvastatin in the hearts of diabetic rats	[21]
		Chronic atorvastatin study plus IPost		10 mg/kg daily for plus IPost	2 weeks	eNOS phosphorylation Akt inhibition No change in the infarct size and myocardial dysfunction	
Animal study	Male Wistar diabetic rats (40 mg/kg STZ, i.p.), nondiabetic rats	*N*: 6	Atorvastatin	10 mg/kg	24 hr	Improvement in CD44 and caspase-3 expression and oxidative stress and kidney function biomarkers in both groups	[22]
Animal study	Injection of low-dose STZ and HFD to male Sprague-Dawley rats	*N*: 5	Atorvastatin	A single 10 mg/day	No	↑ hepatic CYP3a and OATP1b2 expression ↓ CYP3a and OATP1a5 expression in the intestine	[23]
Animal and cellular study	Diabetic rats induced by STZ (35 mg/kg) and HFD	*N*: 25	Atorvastatin	10 mg/kg, 20 mg/kg, and 40 mg/kg	10 days	10 mg/kg and 20 mg/kg of atorvastatin caused severe hepatotoxicity 40 mg/kg of atorvastatin was lethal Upregulations of SLCO1B1 and CYP3A4 in HepG2 cells ROS production	[19, 20]
	HepG2 cells			2, 10, and 30 *μ*M			
Case-control study	Hyperlipidemic HD Caucasian patients	10/14	Simvastatin	A single 10 mg/day	6 months	More lowered anti-inflammatory effects in nondiabetic patients	[24]
Case-control study	Diabetic patients with low-density lipoprotein cholesterol: 50 to 125 mg/dL	4933 (27%) patients with DM	Placebo/simvastatin	40 mg	7 years	↓ low-density lipoprotein cholesterol in diabetic patients ↓ myocardial infarction and ischemic stroke in diabetic patients ↑ NSTE ACS in diabetic patients	[25]
Retrospective propensity score-matched cohort study	Patients with or without diabetes	545 diabetic and 1292 nondiabetic	Simvastatin	20 mg/kg	From January 2005 to June 2015	↑ the incidence rate of mortality in diabetic patients	[26]
Animal study	Male Wistar diabetic rats (35 mg/kg, i.p.)	*N*: 6	Simvastatin	20 mg/kg, p.o. 2 mg/kg, i.v.	Single dose	↓ simvastatin and simvastatin acid AUC ↑ Cyp3a function and simvastatin metabolism ↑ Cyp3a1 and Oatp2 mRNA expression	[27]
Cellular study	Primary hepatocytes of diabetic rats						
Prospective study	Patients who had undergone PCI	202 diabetic (120 on fluvastatin, 82 placebos)	Fluvastatin	40 mg twice daily	3 to 4 years	↓ MACE risk in diabetic patients	[28]
		1475 nondiabetic (724 on fluvastatin, 751 on placebo)					
Animal study	Male Wistar diabetic rats (50 mg/kg STZ, i.p.) and nondiabetic rats	*N* = 6	Fluvastatin	5 mg/kg	24 hr	Alteration in pharmacokinetics of fluvastatin because of diabetes in a stereoselective manner	[29]
Animal study	Male Wistar diabetic rats (50 mg/kg STZ, i.p.)	*N*: 4	Pravastatin	5 mg/kg i.v.	—	↑ expression of oatp2 ↓ pravastatin transportation into hepatocytes ↓ plasma concentration of pravastatin in diabetic rats. ↓ MRP2 expression ↓ transportation of pravastatin to bile ↓ biliary excretion	[30]
Animal study	Male Wistar diabetic rats (35 mg/kg, b.w., i.p.)	*N*: 4	Pravastatin	10 mg/day	28 days	↑ serum pravastatin concentration	[31]
Prospective study	Consecutive AMI patients (diabetic or nondiabetic) underwent PCI	802 patients	Pitavastatin	2 mg/day	12 months	Diabetes was recognized as an independent predictor of TVR-MACE	[32]
Prospective study	Elderly patients with or without type 2 diabetes and hypertension	80 patients	Pitavastatin simvastatin	Pitavastatin: 1-2 mg/d Simvastatin: 10-20 mg/d	2, 4, 8 weeks	Alike decrement in blood lipid and liver dysfunction in both groups	[33]
Prospective study	Chinese diabetic and nondiabetic patients with acute ischemic stroke history	It varies based on the types of models in the study	Any type of statins	Any dosages of statins	3 months, 1 year	No relationship between statin therapy and stroke recurrence in patients with diabetes A remarkable association with lower stroke recurrence in patients without diabetes	[34]
Prospective study	Diabetic and nondiabetic patients affected coronary artery disease who undergone serial optical coherence tomography imaging	54 plaques in 41 diabetic patients and 63 plaques in 49 nondiabetic patients	Any type of statins	Any dosages of statins	1 year	↑ minimum FCT in both groups ↓ lipid index in both groups The same vascular response to statin treatment in both groups	[35]
Retrospective cohort study	Patients with and without diabetes aged 75 years or more without a history of atherosclerotic CVD and statin use or they were statin new users	46864 people aged 75 years or more	Any type of statins	Any dosages of statins	2006-15	In nondiabetic patients: ↑ atherosclerotic CVD incidence until more than the proposed risk thresholds for statin treatment In patients with diabetes: ↓ in atherosclerotic CVD incidence and mortality	[36]

ACS: acute coronary syndrome; ΔLDL: LDL-C; ΔPAV: percent atheroma volume change; STZ: streptozotocin; CYP: cytochrome P450; AUC: area under the curve; eNOS: endothelial nitric oxide synthase; HFD: high-fat diet; SLCO1B1: solute carrier organic anion transporter family member 1B1; HepG2 cells: liver hepatocellular carcinoma; ROS: reactive oxygen species; HD: hemodialysis; NSTE ACS: non-ST segment elevation acute coronary syndromes; MACE: major adverse cardiovascular events; MRP2: multidrug resistance-associated protein 2; AMI: acute myocardial infarction; PCI: percutaneous coronary intervention; FCT: fibrous cap thickness; CVD: cardiovascular disease.

## Data Availability

This is a narrative review that does not have data.
